# The Physiological Action of Alcohol

**Published:** 1882-02

**Authors:** John S. Lynch

**Affiliations:** Professor of Principles and Practice of Medicine and Diseases of the Heart and Lungs, College of Physicians and Surgeons, Baltimore


*FOR CONTENTS OF THIS NUMBER SEE PAGE 144.*

*THE*

### Independent Practitioner.

VOL. III.——*February 1882.*——No. 2.

### ORIGINAL COMMUNICATIONS.

*We are not responsible for the opinions expressed by contributors.*

ARTICLE I.

### THE PHYSIOLOGICAL ACTION OF ALCOHOL.

BY JOHN S. LYNCH, M. D.,
Professor of Principles and Practice of Medicine and Diseases of the Heart and Lungs, College of Physicians and Surgeons, Baltimore.

The physiological action of alcohol presents, when closely observed and analyzed, some apparently singularly contradictory phenomena. Long regarded as a direct arterial and cerebral stimulant, ingenious theories were formed to account for its paralytic action when taken in inordinate quantities. Since Richardson and others, however, have proved that it is certainly sedative in its action upon the sympathetic nerves, and probably upon the cerebro-spinal system also, nearly all the phenomena of alcoholic intoxication become readily explicable without calling in the aid of such absurd expressions as "super excitement," "over stimulation," &c. It is now clearly understood that the first effects of alcohol, such as increase of the frequency and force of the pulse, are due not to increased force in the contractions of the heart, but to diminished resistance to the blood current in the arterioles and capillaries. It is the brake removed from the car wheel, or the pendulum from the clock. In each case the *motion* is increased, though the propelling force remains unaltered. In this as in almost all other poisons, and probably in all the so-called morbific agents, the depressing effects of the drug are felt by the brain and spinal system last. Alcohol therefore taken in moderate quantities would seem to produce the apparently opposite effects of paralyzing the vaso-motor branches of the sympathetic, while stimulating the brain and spinal marrow, producing an increase of power in the voluntary muscles and exaltation of all those intellectual phenomena conveniently expressed by the word cerebration. But this increase of intellectual vigor is due to the indirectly stimulant effect of the hyperemia, and not to any directly stimulating action of the poison upon the brain itself, or to any increase of the tissue changes, which modern pathologists assert to be the source of calorification. The blood is the proper nutrient and stimulant of all the tissues, and hence the increased power of the muscular contractions does not depend so much upon the enlarged innervation as to the increased contractility of the muscular fibres themselves contingent upon the larger amount of blood circulating through their capillaries. If the quantity of alcohol be now considerably increased, we find that the symptoms of paralysis of the voluntary nerves are added to that of the ganglionic system. The movements of the voluntary muscles become unsteady and beyond the control of the intoxicated person, and sensation is impaired also, sometimes perverted, but always diminished. This paralysis of the spinal nerves seems to begin and progress from the periphery to the centre, attacking first the lower extremities, gradually advancing upwards until it reaches the medulla oblongata, when the pneumogastric becomes involved and both respiration and pulsation cease.

The nerves which supply organs of nutrition and secretion as well as excretion suffer *pari passu* with the vaso-motor nerves and while the usual destructive tissue changes are not entirely arrested, they are undoubtedly diminished, while the elimination of the products of this change is almost entirely suspended. Although, therefore, appetite is lost and digestion impaired during a prolonged debauch, yet the body does not seem to waste nor does the victim feel the sensation of hunger, probably because first, the sensory filaments of the nerves are paralyzed along with the trophic and motor filaments, and second, because there is no diminution of the solid elements of the blood. The vessels are still filled with a fluid, which has not given up to the glandular organs the effete material which it has absorbed in its circuit, and which should have been excreted by them. The urea, creatin, hematin, cholesterin, &c., which should have been thrown off through their proper emunctories remain in the blood or are reabsorbed from the bladder, intestines and gall ducts and hence in consequence of inattention to the calls of nature, we have, when the debauch is greatly protracted, a peculiar and remarkable train of phenomena. The skin becomes dingy, muddy and sodden ; the breath gives out a sickly, fetid, ammoniacal smell, and the whole body emits an exceedingly offensive stercoracious or fecal odor, called by old topers familiar with it the "rotten whiskey smell." The continued accumulation in the blood of these effete elements along with the prolonged hyperemia of the cerebral capillaries soon produces the effects we would look for from this state of things, namely delirium, convulsions, stupor and death—death from so-called *mania a potu*, but in reality death from blood poisoning from uremia or ammonemia. If, however, the alcohol is withdrawn, either by necessity or design before this condition is reached, we find another and quite different train of symptoms. The nervous system being relieved of the paralyzing effect of the alcohol, the eliminative organs resume their wonted activity. The urine which was before of low, now assumes a high specific gravity, and instead of being perfectly limpid, becomes highly colored and loaded with deposits. Perspiration becomes active and is also highly charged with various salts, and in fine all the organs of excretion evince a degree of activity commensurate with the accumulated debris they are called upon to remove, and there is thrown off by them in a few hours an amount of material, which probably under ordinary circumstances would have required days to remove. But this removal of such an enormous amount of salts and proteid substances from the blood and the sudden contraction of the capillaries, consequent upon resumption of power in the vaso-motor nerves, produces its inevitable consequence, an acute attack of anemia, especially of the brain and cord, just as that condition follows a severe hemorrhage, or a protracted fever in which tissue destruction is rapid and nutrition arrested. This anemia announces itself by tremor of the muscles (subsultus) and perverted sensation and delirium and hallucination, and sleeplessness. This train of symptoms we find to be common to all attacks of anemia of the brain, whether caused by excessive hemorrhage, prolonged inanition, protracted fever, as typhoid, typhus, &c., or excessive indulgence in alcoholic stimulants. It would follow then that the best way to treat delirium tremens *ought* to be, to give an abundance of nutritious food, and in many cases we know that this is amply sufficient to effect a cure in a few days, but it unfortunately happens that in a large majority of these cases, the digestive organs have been so impaired by either the directly irritant effect of the alcohol, or the indirect impairment of function, shared by all the organs from the anemic state of the blood and paralysis of the trophic nerves, that digestion and assimilation become very slow, if not entirely arrested, and that but a very limited quantity of food can be taken, and sometimes even absolute rest is required for a time to enable these organs to regain their lost powers. Besides, the wakefulness causes a constant activity of the cerebral functions, and this implies a constant destruction of nerve tissue; for cerebration can no more be carried on, except at the expense of the nerve cells or ganglia than can muscular contraction but at the expense of the sarcous elements of the muscles themselves. Arrest of this mental activity becomes therefore a highly important consideration, and of course this can only be done by the induction of sleep; but a consideration of the means of accomplishing this does not come within the scope of this paper.

It may be asked in view of this theory, how sleep alone is competent to the cure of this state of things, as it not unfrequently does. The answer is, that all the tissues when relieved of the wear, incident to their function, very rapidly assimilate from the blood the material necessary to their complete repair, and even when no pabulum is furnished from without, an abundant source of nutrition is generally found within the system in the form of fat which is generally abundantly stored up, apparently to meet such contingencies. Sleep having suspended cerebration, there is no longer any destruction of nerve cells, and those that are lost are rapidly replaced either from the food taken or the source just alluded to.

The influence of alcohol on the function of calorification seems to be as contradictory and paradoxical as is its action upon the nervous system. It had always been assumed, until the thermometer was introduced into clinical observations, that alcohol increased the body heat. This error was founded on the well known fact, that the hands and feet and the peripheral surface of the body generally *do* become actually hotter than natural, under the influence of alcohol. This fact is due to the vaso-motor paralysis alluded to, by which an increase of volume, as well as increased rapidity of the blood current brings more heat to the surface than usual. The peripheral nerves being bathed in this warm blood, fresh from lungs and heart, causes that sensation of glow and warmth, which in cold weather is so grateful to the toper after "taking a drink." But this glow and warmth of the skin is obtained only at the expense of the general stock of caloric evolved in the system; and as the rate of radiation depends much upon the relative heat of the radiating body and the surrounding media, so under the influence of alcohol the rate of radiation and consequent heat loss is very greatly increased by the increased heat of the skin. Under very excessive use of the drug the temperature falls below 90°, and Dr. J. H. Branham, of Baltimore, reports a case of intoxication and the effects of cold in which the temperature was below 83° Fahrenheit, with recovery.

But increased radiation is not the only mode in which alcohol reduces temperature; for if it were we would witness the same phenomenon when the temperature of the skin is greatly elevated in fever. This we never do. There must then be some other factor at work in reducing the general temperature. What is that factor? Do there really exist calorific nerve-centres as has been supposed, and does alcohol paralyze these along with all other ganglia? This explanation can not be accepted in the face of well known physiological and pathological facts. If there be calorific centres all clinical facts go to prove that they are inhibitive and not stimulative. How else can we possibly explain the excessive elevation of temperature sometimes seen in inflammation and injuries of the cord; and especially how can we account for the continued rise of temperature after death in some of the fevers?

This theory, therefore, can not be accepted as true.

The true explanation of the phenomenon of reduced temperature under the influence of large doses of alcohol, is probably the one which, first taught by myself (so far as I know) in my lectures in the College of Physicians and Surgeons during the winter of 1873–4, has since been adopted by Richardson, of London, and very generally accepted by pathologists every where; it is this:—

Alcohol being a simple hydro-carbon, has a stronger affinity for oxygen than the more complex fatty and nitrogenous substances which ordinarily constitute the fuel whose combustion supplies animal heat. The blood being saturated with alcohol, the oxygen seizes upon *it* instead of the latter substances; and it is in this way that alcohol becomes an arrester of waste, and indirectly a conservator of tissue and strength as mentioned incidentally above. But the oxidation of this simple hydro-carbon is not attended with the evolution of so much heat as could be produced by the combustion of those other more complex bodies mentioned, and consequently the supply of heat is diminished in the interior of the organism. But we have seen that the heat of the surface is increased, and this increases radiation or heat loss. It is in both ways, therefore, that alcohol reduces the temperature when taken in large quantities, and I was correct in describing its action as apparently contradictory and paradoxical; since it heats up the skin while it lowers the temperature of the heart and lungs.

The conclusions I arrive at then from these facts and hypotheses are:

1st. Alcohol is a direct sedative to the nervous system, and only increases the force and frequency of the pulse indirectly by diminishing capillary and arterial tension.

2nd. It does not increase digestion or assimilation, but diminishes both.

3rd. It probably primarily increases tissue changes when taken in moderate quantities, but causes retention in the blood of the products of these changes by diminishing the activity of all the eliminating organs, when the poison is administered in large or often repeated doses.

4th. It rapidly reduces the bodily temperature by diminishing the evolution of heat while increasing radiation or heat loss.

5th. It is probable, from the experiments and observations of Harley, Bernard and others, that it occasionally increases the nutrition of the body by stimulating the glycogenic function of the liver when small doses are taken, but in large quantities it diminishes it by producing diabetes, polydipsia, or polyuria.

